# Binge-Like, Naloxone-Sensitive, Voluntary Ethanol Intake at Adolescence Is Greater Than at Adulthood, but Does Not Exacerbate Subsequent Two-Bottle Choice Drinking

**DOI:** 10.3389/fnbeh.2020.00050

**Published:** 2020-04-09

**Authors:** Agustín Salguero, Andrea Suarez, Maribel Luque, L. Ruiz-Leyva, Cruz Miguel Cendán, Ignacio Morón, Ricardo Marcos Pautassi

**Affiliations:** ^1^Instituto de Investigación Médica M. y M. Ferreyra, INIMEC–CONICET-Universidad Nacional de Córdoba, Córdoba, Argentina; ^2^Facultad de Psicología, Universidad Nacional de Córdoba, Córdoba, Argentina; ^3^Department of Pharmacology, Faculty of Medicine, University of Granada, Granada, Spain; ^4^Institute of Neuroscience, Biomedical Research Center (CIBM), University of Granada, Granada, Spain; ^5^Biosanitary Research Institute (IBS), University Hospital Complex of Granada, Granada, Spain; ^6^Department of Psychobiology and Research Center for Mind, Brain, and Behavior (CIMCYC), University of Granada, Faculty of Psychology, Granada, Spain

**Keywords:** ethanol, Wistar, binge exposure, naloxone, adolescence

## Abstract

The present study assessed the effects of ethanol exposure during adolescence or adulthood. We exposed Wistar rats, males or females, to self-administered 8–10% (v/v) ethanol (BINGE group) during the first 2 h of the dark cycle, three times a week (Monday, Wednesday, and Friday) during postnatal days (PDs) 32–54 or 72–94 (adolescent and adults, respectively). During this period, controls were only handled, and a third (IP) condition was given ethanol intraperitoneal administrations, three times a week (Monday, Wednesday, and Friday), at doses that matched those self-administered by the BINGE group. The rats were tested for ethanol intake and preference in a two-bottle (24 h long) choice test, shortly before (PD 30 or 70) and shortly after (PD 56 or 96) exposure to the binge or intraperitoneal protocol; and then tested for free-choice drinking during late adulthood (PDs 120–139) in intermittent two-bottle intake tests. Binge drinking was significantly greater in adolescents vs. adults, and was blocked by naloxone (5.0 mg/kg) administered immediately before the binge session. Mean blood ethanol levels (mg/dl) at termination of binge session 3 were 60.82 ± 22.39. Ethanol exposure at adolescence, but not at adulthood, significantly reduced exploration of an open field-like chamber and significantly increased shelter-seeking behavior in the multivariate concentric square field. The rats that had been initially exposed to ethanol at adolescence drank, during the intake tests conducted at adulthood, significantly more than those that had their first experience with ethanol at adulthood, an effect that was similar among BINGE, IP and control groups. The study indicates that binge ethanol drinking is greater in adolescent that in adults and is associated with heightened ethanol intake at adulthood. Preventing alcohol access to adolescents should reduce the likelihood of problematic alcohol use or alcohol-related consequences.

## Introduction

Alcohol (subsequently also referred to as ethanol in the context of pre-clinical studies) use is highly prevalent during adolescence in most western countries. Illustrating this point, a study (Pilatti et al., 2017) reported that, in the 6 months preceding data collection, 70% of a sample of college students from Argentina (*n* > 4,000, average age: 19 years) ingested 6–7 drinks of alcohol on the same drinking occasion; and 55% consumed 4–5 drinks in 2≤ h (a pattern known as “binge drinking”). Approximately 33 and 20% reported these patterns, respectively, on a weekly basis, and the average consumption on Saturdays was 5–8 drinks, reaching 10 in those at-risk for exhibiting family history of alcohol problems. These consumption patterns can yield immediate negative consequences [domestic accidents, increased risk of engaging in interpersonal violence or in unsafe sexual practices (Pilatti et al., 2014; Wicki et al., 2018)], but they are also associated with heightened risk of developing alcohol use disorders later in life (Pedersen and Skrondal, 1998; Hingson and Zha, 2009). This is, the earlier the first experience with alcohol, the greater the odds of high-risk alcohol consumption (Rial Boubeta et al., 2018) or alcohol use disorders, although it not yet clear if these two events are causally related, or if they are dependent on a third factor [e.g., genetic predisposition (Buchmann et al., 2009)].

This “early debut” effect (i.e., the early the onset of alcohol use, the greater the later problematic use of alcohol) has been shown across cultures, and is illustrated by a seminal clinical work (DeWit et al., 2000), that reported 16% of alcohol dependence in those who began drinking at 11–12 years, but only 1% in those who started at age 19. Our studies (Pilatti et al., 2013) have also indicated that college students who started drinking alcohol at ≤15 years exhibit significantly more alcohol use and drunkenness than those who had their first contact with the drug after age 15. Perhaps more important, another clinical study has recently shown (Vera et al., 2019) that the initial contact with alcohol is not as relevant, as a predictive milestone for subsequent problematic substance use, as the first intoxication or drunkenness episode. In the latter work a greater number of alcohol-related negative consequences after an early onset of drinking was observed only in participants that already had experienced an intoxication or drunkenness episode. Level of alcohol-related problems was low and not affected by age of drinking onset, in those that were drunkenness naïve. Another study (Kuntsche et al., 2013) indicated that early drunkenness, but not early drinking, predicted several adolescent problem behaviors. This, together with the high prevalence of binge drinking in adolescents, suggests that pre-clinical models of “early alcohol initiation” should focus not so much on mere adolescent drug exposure, but on a type of exposure akin to that of drunkenness. Modeling drunkenness in rats or mice is problematic, as drunkenness is mainly defined by a subjective state. A better alternative is to generate models that induce high levels of ethanol consumption in a short timeframe, compatible with the definition of binge drinking.

The pre-clinical models of the “early alcohol debut” effect have not been consistent as to whether early onset of ethanol use is associated with later heightened ethanol use. The phenomenon has been modeled in rodents by repeatedly exposing rats or mice to high doses of i.p. or i.g. ethanol (e.g., 2.5–3.0 g/kg every 48 h) during the adolescence, which in rodents is usually defined as the period between postnatal days (PD) 28–42 or 60 (Spear, 2000). These experimenter-administered treatments induce, in mice or rats, neuroinflammation (Fernandez-Lizarbe et al., 2009; Pascual et al., 2009), alterations in glutamatergic and dopaminergic transmission (Trantham-Davidson et al., 2017), and also alter the neural pruning of the latter transmitter system (Pascual et al., 2009). They also exert detrimental effects upon the functionality of the hypothalamic-pituitary-adrenal axis (Asimes et al., 2017) and modulate gene expression of histone deacetylase 1 (Lopez-Moreno et al., 2015). These studies are useful to demonstrate persistent changes associated with early exposure to alcohol that induces level of intoxication compatible with or beyond the level that define binge drinking, but they have the limitation of employing routes of administration that exert behavioral and neural effects very different from those induced by the self-administration of the drug. It is not surprising, then, that these models have generated conflicting data. In our laboratory (Fabio et al., 2014) we have observed that only two or five ethanol intubations during the early adolescence of the rat increase the subsequent consumption of ethanol, while the same treatment in adulthood does not exert significant effects, data consistent with that reported by Pascual et al. (2009). However, others did not observe alterations in adult ethanol consumption after forced exposure to vaporized ethanol during the adolescence of the rat (Nentwig et al., 2019). Illustrating the contradictions inherent in these models, both Broadwater et al. (2011) and our laboratory (Pautassi et al., 2015) observed that 6 or 10 i.p. or i.g. administrations of ethanol (2.25–4.0 g/kg every 24 or 48 h) induced chronic tolerance to ethanol or inhibited aversive conditioning toward alcohol in adolescent, but not in adult, rats. However, both studies (Broadwater et al., 2011; Pautassi et al., 2015) reported that the adolescents exposed to ethanol exhibited similar levels of ethanol intake, when evaluated in two-bottle choice tests, than adolescents that had been only exposed to vehicle.

The present study assessed the effects of ethanol exposure during adolescence or young adulthood on two-bottle, free-choice, ethanol drinking. The latter consumption was tested either immediately after termination of ethanol exposure, or in late adulthood, at PDs 120–139. Specifically, we assessed short- and long-term effects of early or late age of onset of alcohol drinking, in an animal model – adapted from the drinking-in-the-dark (DID) paradigm (Boehm et al., 2008; Rodriguez-Ortega et al., 2019) – that mimics a binge pattern of alcohol consumption. In other words, the aim was not just to model early onset of drinking, but to induce levels of drinking akin to those found during a binge drinking episode, whose threshold is often defined at 80 mg/dl (e.g., Hosova and Spear, 2017).

More in detail, adult – male or female – rats self-administered 8–10% (v/v) ethanol during the first 2 h of the dark cycle, three times a week for 4 weeks. Controls were only handled and a third condition was given ethanol i.p. administrations, three times a week for 4 weeks, at doses that matched those self-administered. The rats were tested for ethanol intake and preference in a two-bottle (24 h long) choice test, shortly before (i.e., baseline) and shortly after exposure to the binge protocol; and then were tested for 3 weeks during late adulthood (PDs 120–139) in intermittent two-bottle ethanol intake tests. The effects of the binge-like exposure during adolescence or adulthood upon anxiety response, shelter seeking/risk-taking and recognition memory were also tested, via the light-dark box (LDB) test (Acevedo et al., 2014), the multivariate concentric square field (MSCF) test (Roman and Colombo, 2009; Ekmark-Lewen et al., 2010) and the novel object recognition (NOR) test (Antunes and Biala, 2012), respectively. Experiment 2 assessed if treatment with the opioid antagonist naloxone could inhibit ethanol binge drinking at adolescence, and measured the blood ethanol levels (BELs) achieved during the binge procedure.

## Materials and Methods

### Experimental Design and Subjects

**FIGURE 1:**
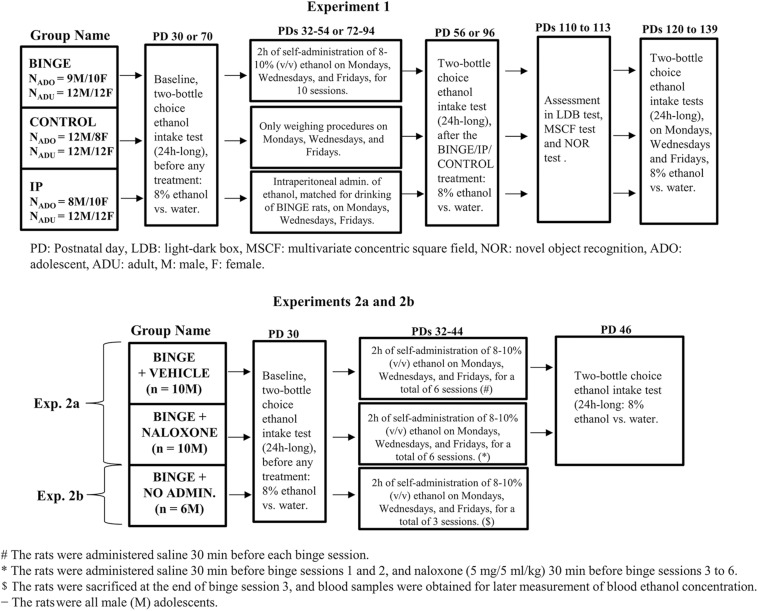
Schematic representation, including experimental timelines and sample size in each group, of the methods for the analysis of short- and long-term effects of ethanol binge drinking, in Experiment 1 and in Experiments 2a and 2b (upper and lower sections, respectively). In Experiment 1 the adolescent or adult rats, males or females, self-administered 8–10% (v/v) ethanol during the first 2 h of the dark cycle, three times a week for 4 weeks (BINGE group). Rats in the CONTROL group were only handled, and those in the IP condition were given ethanol intraperitoneal administrations, three times a week for 4 weeks, at doses that matched those self-administered by same-sex counterparts in the BINGE condition. The rats were tested for ethanol intake and preference in two-bottle (24 h long) choice tests, shortly before (i.e., baseline) and shortly after exposure to the binge protocol; and then again at late adulthood [postnatal days (PDs) 120–139]. The rats were tested on PDs 110–113 in the light-dark box (LDB) test, the multivariate concentric square field (MSCF) test and the novel object recognition (NOR) test. Experiment 2a repeated the BINGE conditions in male adolescents and assessed if treatment with the opioid antagonist naloxone, administered 30 min before binge sessions 3–6, inhibited ethanol binge drinking at adolescence and later ethanol consumption in a two-bottle choice intake test. Experiment 2b measured the blood ethanol concentrations achieved during the binge procedure, in 6 male rats that were sacrificed at the end of binge session 3.

A total of 155 Wistar rats (129 in Experiment 1, 26 in Experiment 2) were employed. A 2 (age of first contact with ethanol: adulthood vs. adolescence) × 2 (sex: male vs. female) × 3 (mode of early ethanol exposure: binge-like self-administration, i.p. administrations or control, BINGE, IP and CONTROL groups, respectively) factorial was employed in Experiment 1, with 8–12 Wistar rats in each group. Experiment 2a assessed the effects of naloxone upon binge ethanol drinking in 10 male rats, whereas another group of 10 males was administered vehicle. Experiment 2b exposed 6 male adolescents to three sessions of binge drinking. These rats, which were not administered naloxone or vehicle, were sacrificed at the end of the third binge session. Blood samples were obtained and subsequently processed to yield a measure of BELs achieved during the 2 h binge session. A schematic representation of the experiments, containing a detailed account of the number of animals in each experimental group can be found in Figure 1.

The rats were born and reared at one of the vivarium of the Instituto de Investigación Médica Mercedes y Martín Ferreyra (INIMEC-CONICET-UNC; Córdoba, Argentina), a producer of specific pathogen free rats, and were derived from 20 dams. As per policy of the vivarium, all the litters are culled on PD1 to 10 rats (5 males, 5 females, whenever possible). Lights were turned on at 645 and turned off at 1845. Litter effects were controlled by not including more than one male and one female from each litter to each group. Weaning was performed at PD 21, and from that on the rats were housed in same-sex pairs. The procedures complied with the Declaration of Helsinki, the ARRIVE guidelines, and the Guide for the Care and Use of Laboratory Animals promulgated by the NIH and the EU. The procedures were certified by the Institutional Animal Care and Use Committee at INIMEC-CONICET-UNC.

### Repeated Exposure to Ethanol on Adolescence or Early Adulthood (Exp. 1)

The rats, males and females, were housed into same-sex groups of two and weighed every day. The day before each BINGE session or IP administration the rats in groups BINGE and IP were given 50% of the water they usually consumed, as a means to promote ethanol consumption in the upcoming binge session or, in the case of the IP group, to keep hydration conditions similar to those in the BINGE group.

For 4 weeks (Adolescents: PDs 32–54, Adults: PDs 72–94) the BINGE rats were exposed on Mondays, Wednesdays and Fridays (except the first Monday of the 1st week and the last Friday of the 4th week, see next section) to a bottle of 8% (first two sessions) or 10% ethanol (third and subsequent session) between 1900 and 2100 h.; this is, 15 min after the beginning of the dark cycle. Subsequently, the rats in the IP group were given an intraperitoneal administration of ethanol (20% v/v, volume of administration: 0.01 ml/g of body weight) whose dose was matched for the level of ethanol ingestion exhibited by their same-sex counterparts of the binge group. This is, the rats in the IP group were exposed to ethanol three times a week for 4 weeks, mirroring the schedule of ethanol exposure of the BINGE group. The inclusion of the IP group, which could be referred to as a “matched condition,” was meant to determine whether any effect of early alcohol exposure upon later ethanol drinking during late adulthood was a consequence of total ethanol exposure or if the mode of exposure (self- vs. experimenter-administered) was the key factor. Control rats were left undisturbed, except for the daily weighing procedure. Other than in the times specified, BINGE and IP rats were given water ad-libitum.

The binge protocol was designed by combining well-established preclinical protocols from our group (Wille-Bille et al., 2017) to assess every-other-day ethanol drinking and those of the daily limited-access ethanol intake model referred to as DID (Boehm et al., 2008). At the beginning of each binge session the housing chambers were divided into two sections, by a Plexiglas separator, and each animal occupied half of the cage. Thus, rats in the BINGE group could smell (but not touch) each other, reducing potential isolation effects. Each section was equipped with one glass bottle, with rubber caps with stainless a steel spout with round tip. The rats were exposed to a bottle filled with 8% or 10% alcohol (vehicle: tap water). Spillage/leaking was accounted for by having a control bottle in an empty cage. The rats had *ad libitum* access to food during the 2 h session. After the 2 h (that is, at 2100) the bottle was replaced by a water bottle. The ethanol bottle was weighed before and after the session and these scores were used to calculate the g/kg of alcohol consumed.

### Two-Bottle Choice Ethanol Intake Tests Conducted Immediately Before or Immediately After Binge Exposure (Exp. 1)

One of the aims of the study was to evaluate free-choice ethanol drinking immediately after termination of the chronic, binge-like ethanol exposure. Thus, the rats – males and females – were assessed on a two-bottle, 24 h free-choice, test on PD 30 or 70 (adolescent or adult groups, baseline pre-test before any treatment) and on PD 56 or 96 (i.e., post-test after termination of the binge-like exposure or the intraperitoneal administrations).

The two-bottle choice tests were conducted following procedures described previously (Fernandez et al., 2017). Briefly, at 900 a Plexiglas divider was used to individually house each rats in half of the homecage. A special lid allowed equipping each section with two bottles and *ad libitum* access to food. One of the bottles contained water, the other was filled with 8% ethanol (v/v). The bottles were weighed before and after each session, and the difference was used to calculate ethanol intake (g/kg) and the percent preference of ethanol intake [(ethanol consumption/overall fluid consumption) × 100].

### Behavioral Assessments Following Exposure to Binge Ethanol (Exp. 1)

At late adulthood all the rats were assessed for anxiety response, shelter-seeking and risk taking and overall exploratory patterns, and for recognition memory. These variables were measured via LDB test (Acevedo et al., 2014), the MSCF test (Roman and Colombo, 2009; Ekmark-Lewen et al., 2010) and the NOR test (Antunes and Biala, 2012), respectively. As it will be described in this section, the MSCF also serves to measure, along with other behaviors, anxiety-like responses. The order of the LDB and the MSCF tests was randomly counterbalanced and took place on PDs 110 or 111, the NOR took place on PDs 112 and 113.

The LDB test was that described in Wille-Bille et al. (2018). Briefly, we employed a rectangular apparatus featuring two sections [one white (24.5 cm × 25 cm × 25 cm, 400 lux illumination), the other black (17.5 cm × 25 cm × 25 cm, 0 lux illumination)] connected by an opening at floor level. Testing lasted 5 min and began by placing the rats in one of the corners of the white section facing the wall. Time spent in the white compartment, latency (s) to first exit the white compartment and number of transfers between compartments were measured.

The MSCF test (Roman and Colombo, 2009) is a 20-min long assay that allows recording several exploratory behaviors simultaneously (Karlsson and Roman, 2016). The rats are gently introduced in a square-shaped apparatus (48 cm × 48 cm) featuring an open-field like center square (OF, the starting area), a highly illuminated (650 lux) area featuring a ramp (RAMP, 12 cm × 10 cm, 20° incline) that lead to a metallic structure (the BRIDGE, 30 cm × 10 cm, 650 lux) that prompted exploration, a dark and enclosed area that evokes shelter-seeking behavior (SHEL, 0 LUX), 3 connecting corridors or passages (P, 20–30 lux), and a small section similar to the SHELL yet slightly more illuminated (30 lux) and inaccessible by regular horizontal locomotion. Access to the latter area, referred to as challenge area (CHA) required performing a jump through an elevated hole. Time spent in SHELL is usually considered an indicator of anxiety response (Wille-Bille et al., 2018). The test was filmed and time spent and number of entries in each area was recorded offline using JWatcher 1.0 (Blumstein and Daniel, 2007). Total number of entries into the different sections was considered an index of overall motor activity.

The novel object recognition (NOR) test assesses short-term memory (Vogel-Ciernia and Wood, 2014). We employed an open-field like, squared-shaped, arena made of Plexiglas (50cm × 50cm × 50cm), equipped with photo sensors that virtually divided the arena into 25 squares. A software (ITCOMM, Córdoba, Argentina) detected, in a minute-by-minute basis, the number of beam breaks. The rats were introduced into the empty arena for 10 min, in a habituation phase (PD112) that also served to analyze exploratory patterns in an open field like novel environment. A day later (familiarization phase) the rats were left to explore the arena for 5 min, which was now equipped with two identical objects (i.e., A and A’, opaque glass flasks) in the upper corners. Twenty minutes later one of the objects was replaced by a new object (B, taller and slightly clearly colored compared to A/A’) and the rats had another 5-min trial (testing phase) in which they freely explored the arena. The tests were videotaped and analyzed. Time spent in close proximity to the objects was measured, in a minute-by-minute basis, during the familiarization and testing phase. Time spent in proximity to the new object B at the testing phase was considered an indicator of short-term memory (Vogel-Ciernia and Wood, 2014). The objects were selected on the basis of pilot studies that indicated that rats did not have innate preferences for the objects later designated as familiar or novel.

### Two-Bottle Choice Ethanol Intake Tests Conducted at Late Adulthood (Exp. 1)

Another aim of the study was to evaluate the long-term effects of ethanol exposure during adolescence or adulthood, after imposing a relatively long time between the last exposure to binge ethanol, and when both groups of rats (i.e., those with “early” or “late” onset of alcohol use) were equated in terms of age of testing. Therefore, all the rats were tested for ethanol intake for 3 weeks at late adulthood (PDs 120–139) using the two-bottle ethanol intake test described in section “Two-Bottle Choice Ethanol Intake Tests Conducted Immediately Before or Immediately After Binge Exposure (Exp. 1).” These 24 h tests were conducted intermittently, on Mondays, Wednesdays and Fridays, for a total of nine sessions.

### Naloxone Administration (Exp. 2a) and Measurement of BELs Achieved During the Binge-Like Protocol (Exp. 2b)

Experiment 2a replicated, in 26 adolescent males, the baseline and post-test two-bottle choice measurements of ethanol intake, and binge exposure sessions, of Exp 1.

Specifically, in Experiment 2a the rats (*n* = 20) were administered saline 30 min prior to the beginning of binge sessions 1 and 2. From session 3 to session 6, half of the rats received naloxone (Sigma Aldrich, Buenos Aires, Argentina; subcutaneous, 5 mg/5 ml/kg) and the remaining received saline. Naloxone dose and timing of administration were selected based in prior work demonstrating its effectiveness to reduce ethanol self-administration. The six remaining rats were not administered naloxone or vehicle before the binge sessions. At the end of binge session 3 these rats were sacrificed through decapitation and trunk blood samples (2-ml samples) were obtained using a heparinized capillary tube. The samples were centrifuged at high speed (15 min/3,000 rpm) and the vials containing the plasma phase were stored at −70°C for later analysis. BELs were expressed as milligrams of ethanol per deciliter of blood (mg/dL) and assessed via a colorimetric enzymatic method. Specifically, the method was based on the action of the enzyme alcohol dehydrogenase, which uses the oxidized form of the nicotinamide adenine dinucleotide (NAD+) as a cofactor, which is then reduced to NADH. This reduction generates an absorbance increase that is measured at 340 nm. These measurements had a precision/accuracy of ±4 mg/dL and were conducted at LACE labs (Córdoba, Argentina), using a COBAS6000 (Roche, Basel, Switzerland) apparatus.

### Statistical Analysis

The variables measured were first checked for normality and homogeneity of variance, to assure the appropriateness of using parametric statistics. Ethanol intake scores (g/kg) during the binge sessions of Experiment 1 were assessed using repeated measures (RM) Analyses of Variance (ANOVAs) that considered age (adolescence, adulthood), and sex (male, female) as between factors and day of assessment (intake sessions 1–10) as the within-measure. Similar RM ANOVAs were used to analyze ethanol intake scores (g/kg and % preference) and water intake (ml/100 g of body weight) at the two-bottle choice ethanol intake tests conducted before and after the binge sessions. The latter ANOVAs also included mode of ethanol exposure (ethanol exposure via self-administration or i.p. injections, or non-exposed controls: groups BINGE, I.P. and CONTROL, respectively) as a between-subjects factor.

Ethanol intake (g/kg) ingested during the binge protocol of Experiment 2 was analyzed via a 2-way mixed ANOVA, with naloxone administration and day of assessment as the between and within-subject factors, respectively. BELs (mean ± SEM) were correlated with the g/kg ingested on binge session 3 via Pearson’s time-moment correlation (i.e., the association considered the BELs measured in the blood samples and the absolute level of ingestion registered in the session that finalized just before the sacrifice).

The variables measured in the LDB test (latency to enter the black section, time spent in the white section and number of transfers) and in the MCSF test (Experiment 1) were analyzed by separate factorial (sex × mode of ethanol exposure × age at first ethanol exposure) ANOVAs. Activity scores (number of beam breaks in the empty arena) during the habituation phase of the NOR protocol were analyzed via RM ANOVAs (age of first ethanol exposure × sex × mode of ethanol exposure; with minutes 1–10 as repeated measure). Behavioral reactivity during the first and second (test) phase of the NOR protocol were analyzed via RM ANOVAs, that considered sex, mode of exposure and age at first ethanol exposure as between factors, whereas time spent in the vicinity of the objects (i.e., A and A’ or A and B, first phase and test phase, respectively) was the dependent variable. A relative discrimination index (Di) was also calculated [i.e., time spent exploring the novel object minus time spent exploring the familiar object divided by total exploration time (Lueptow, 2017)] and analyzed via a factorial ANOVAs, that considered sex, mode of exposure and age at first ethanol exposure as between factors. Di scores range between -1 and +1, in which a zero score indicates the lack of preference, a negative score indicates more time spent with the familiar object, and a positive score indicates more time spent with the novel object (Antunes and Biala, 2012).

The significant main effects and significant interactions yielded by the ANOVAs were scrutinized via Tukey’s *post hoc* tests or planned comparisons. Planned comparisons were used to analyze significant main effects or interactions comprising between-by-within factors whereas Tukey was used for significant effects involving between-subject factors. There is still debate of which is the best error term for *post hoc* comparisons in significant effects involving within-subject factors. In this scenario, the planned comparisons, which were also employed for a few specific comparisons based on *a-priori* hypotheses, provide a satisfactory compromise between conservativeness and sensitivity (Winer et al., 1991). Data is informed as mean ± SE. Effect sizes of the ANOVAs are described through the partial eta squared (η^2^*p*) and the α level was set at ≤0.05. Effect sizes were interpreted as follows: small (η^2^*p* = 0.01–0.05), medium (η^2^*p* = 0.06–0.13), and large (η^2^*p* = ≥ 0.14) (Lakens, 2013). The statistical analyses were conducted with STATISTICA 8.0 (Tulsa, OK, United States).

## Results

### Ethanol Intake at the Binge-Like, 2 h Sessions of Access to Ethanol (Exp. 1)

**FIGURE 2:**
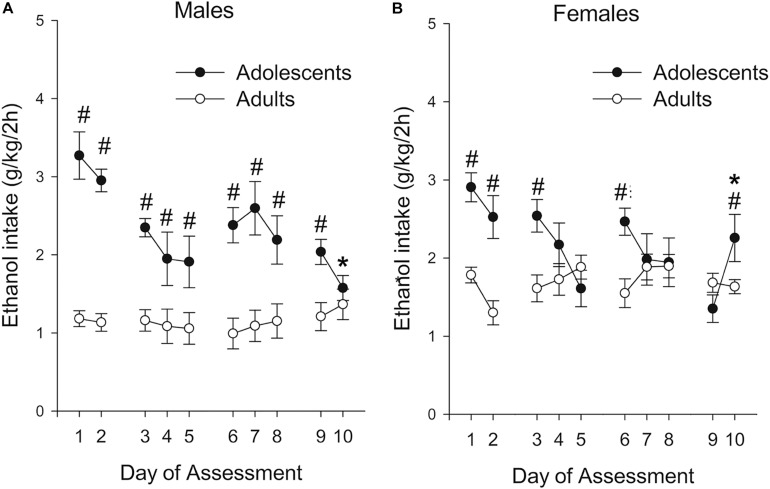
Ethanol intake (g/kg) in male and female Wistar rats (**A** and **B** panels, respectively) as a function of binge intake session (i.e., Day of Assessment 1–10). The rats self-administered 8% (first two sessions) or 10% ethanol (third and subsequent session) during the first 2 h of the dark cycle, three times a week (Monday, Wednesday, and Friday) during postnatal days (PDs) 32–54 or 72–94 (adolescent and adults, respectively). Ethanol drinking was significantly higher in adolescent than in adult rats; an effect that in males achieved significance in all but the last session, whereas in females was significant in sessions 1, 2, 3, 6, and 10. These significant differences are indicated by the numeral (#) sign. Ethanol ingestion in male and female adolescents was significantly greater on Day 1 than on Day 10, an effect indicated by the asterisk (*) sign. Nineteen adolescent (9 males, 10 females) and 24 adult (12 males, 12 females) rats were employed. The data are expressed as mean ± SEM.

As shown in Figure 2, level of ethanol ingestion during the 2 h binge sessions was much greater in adolescents than in adults, with 2- to 3-fold differences between these groups. These differences were particularly noticeable in the first testing days and in males. The ANOVA confirmed these impressions. The analysis of absolute ethanol intake (g/kg) revealed significant main effects of Age and Day of Assessment (*F*_1,39_ = 38.60, *p* ≤ 0.001; η^2^*p* = 0.50 and *F*_9,351_ = 5.87, *p* ≤ 0.001; η^2^*p* = 0.13) as well as significant interactions between Sex and Age (*F*_1,39_ = 6.84, *p* ≤ 0.05; η^2^*p* = 0.15), and between Age and Day of Assessment (*F*_9,351_ = 8.32, *p* ≤ 0.001; η^2^*p* = 0.18). The three-way interaction Sex × Age × Day of Assessment also reached significance, *F*_9,351_ = 2.54, *p* ≤ 0.01; η^2^*p* = 0.06. The planned comparisons indicated that ethanol drinking was significantly higher in adolescent than in adult rats; an effect that in males achieved significance in all but the last session, whereas in females was significant in sessions 1, 2, 3, 6, and 10. The adults kept their level of ethanol ingestion stable across the course of the assessment, whereas adolescents exhibited a progressive decrease, with ethanol ingestion in male and female adolescents being significantly greater on Day 1 than on Day 10. In adult rats, ethanol ingestion was significantly greater in females vs. males across most sessions (i.e., sessions 1, 3, 4, 5, 6, 7, 8 and 9), whereas this variable was not affected by sex in adolescents.

### Ethanol and Water Intake During the Two-Bottle Choice Ethanol Intake Tests Conducted Before and After Binge Exposure (Exp. 1)

**FIGURE 3:**
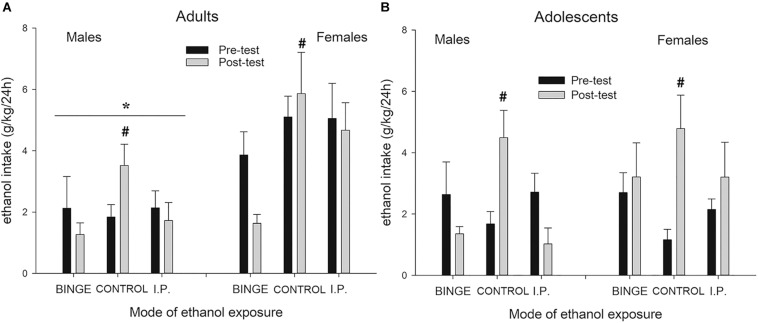
Ethanol intake (g/kg) (**A** and **B** panels, respectively) during two-bottle, 24 h free-choice tests, in male and female Wistar rats as a function of group assignment (BINGE, CONTROL or IP). The rats were assessed on a two-bottle, 24 h free-choice, test on PD 30 or 70 (adolescent or adult groups, pre-test before any treatment) and on PD 56 or PD96 (i.e., post-test after termination of the binge-like exposure). During the BINGE/IP/CONTROL exposure phase the BINGE group underwent 10 sessions in which the rats were exposed to a bottle of 8% (first two sessions) or 10% ethanol (third and subsequent session) between 1900 and 2100 h. After each session the rats in the IP group were given an intraperitoneal administration of ethanol whose dose was matched for the level of ethanol ingestion exhibited by their same-sex counterparts of the binge group. CONTROL rats were undisturbed during the binge/ip exposure phase. The statistical analysis indicated that females drank more than males [an effect indicated by the asterisk (*) sign] and that, at the post-test, the control groups – either male or females, adolescent or adults – drank significantly more ethanol than rats that had been binging or had received i.p. administrations of ethanol. The latter effect is indicated by the hashtag (#) sign. The BINGE group employed 19 adolescent (9 males, 10 females) and 24 adult (12 males, 12 females) rats, the CONTROL group employed 20 adolescent (12 males, 8 females) and 24 adult (12 males, 12 females) rats, and the IP group employed 18 adolescent (8 males, 10 females) and 24 adult (12 males, 12 females) rats. The data are expressed as mean ± SEM.

Figure 3 depicts g/kg of ethanol ingested during the two-bottle choice ethanol intake tests conducted before and after binge exposure. The ANOVA revealed significant main effects of Sex and Mode of early ethanol exposure (*F*_1,114_ = 16.76, *p* ≤ 0.001; η^2^*p* = 0.13 and *F*_2,114_ = 4.39, *p* ≤ 0.05; η^2^*p* = 0.07, respectively). Females rats drank more than did their male counterparts. The interaction between Mode of exposure and Day of Assessment was also significant (*F*_2,114_ = 9.16, *p* ≤ 0.005; η^2^*p* = 0.14). The subsequent Tukey’s *post hoc* tests indicated that all rats, irrespective of the groups they would be assigned during the binge sessions, ingested similar levels of ethanol during the pre-test. On the contrary, at the post-test the Tukey’s *post hoc* tests indicated that the rats that had been binging or had received i.p. administrations of ethanol drank significantly less than CONTROL counterparts.

The ANOVA for ethanol percent preference yielded a pattern (descriptive data not shown) similar to that found for absolute ethanol intake scores. The ANOVA revealed significant main effects of Age and Mode of early ethanol exposure (*F*_1,114_ = 7.36, *p* ≤ 0.01; η^2^*p* = 0.06 and *F*_2,114_ = 3.32, *p* ≤ 0.05; η^2^*p* = 0.06, respectively) and a trend toward a significant effect of Sex, *F*_1,114_ = 3.19, *p* = 0.0766; η^2^*p* = 0.03. Adults exhibited greater ethanol predilection than adolescents. More important, the three-way interaction between Day of assessment, Mode of exposure and Sex (*F*_2,114_ = 3.20, *p* ≤ 0.05; η^2^*p* = 0.05) was also significant. The subsequent planned comparisons indicated that females exhibited fairly similar ethanol predilection in the pre- and post-test, despite the mode of ethanol exposure during the binge phase. On the contrary, control males – but not those given binge or i.p. ethanol exposure – exhibited increased ethanol predilection vs. water in the in the post-test, compared to the pre-test.

The ANOVA for water intake (ml/100 g of body weight) revealed significant main effects of Sex and Age (*F*_1,11__4_ = 12.70, *p* ≤ 0.001; η^2^*p* = 0.10 and *F*_1,11__4_ = 6.89, *p* ≤ 0.01; η^2^*p* = 0.06, respectively). Females and adults drank more water than males and adolescents at the two-bottle choice ethanol intake tests conducted before and after binge exposure, and these effects were not affected by the group in which the rats were assigned, nor there were significant interactions between the factors. Water intake scores can be found in Table 1.

**TABLE 1:** Water (ml/100 g of body weight) ingested during the 24-h two-bottle choice tests.

	Adolescents			Adults		
	Binge	Control	I.P.	Binge	Control	I.P.
**Females**						
Pre-test	8.14 ± 0.97	8.60 ± 2.06	7.21 ± 1.25	12.08 ± 1.37	9.77 ± 1.22	9.63 ± 1.97
Post-test	8.41 ± 0.77	7.85 ± 0.97	9.63 ± 1.36	10.25 ± 0.90	10.11 ± 1.09	10.86 ± 1.48
**Males**						
Pre-test	4.19 ± 0.79	6.82 ± 1.62	6.56 ± 0.96	9.10 ± 1.19	6.07 ± 1.52	7.92 ± 1.50
Post-test	6.00 ± 0.86	6.06 ± 1.3	7.63 ± 2.03	8.23 ± 1.14	7.14 ± 1.25	7.55 ± 1.31

### Behavioral Responsiveness After Exposure to Binge Ethanol (Exp. 1)

Behavioral responsiveness data in the LDB, NOR, and MSCF is presented in Table 2, as a function of age of first ethanol exposure and mode of ethanol exposure. The data is presented collapsed by sex (male, female). As detailed in this section, sex did not exert, for the most part, significant main effects nor was involved in significant interactions. Thus, to facilitate data presentation, data has been collapsed by sex in Table 2.

**TABLE 2:** Behavioral responsiveness measured in each behavioral test.

			Adolescents			Adults		
			Binge	Control	I.P.	Binge	Control	I.P.
LDB	Latency to enter the black side (s)		14.53 ± 2.21*	17.10 ± 2.50*	12.10 ± 1.82*	7.67 ± 1.05	9.87 ± 1.34	11.29 ± 1.16*
	Transitions (freq.)		3.00 ± 0.43	2.05 ± 0.25	2.74 ± 0.35	21.29 ± 4.66*	23.71 ± 4.98*	16.33 ± 4.07*
	Time in white side (s)		34.42 ± 5.26*	31.80 ± 4.79*	41.26 ± 6.07*	14.17 ± 5.33	12.21 ± 4.53	9.21 ± 4.42
NOR PHASE 1	Time spent (s)	A object	13.47 ± 1.93	9.40 ± 1.22	15.59 ± 1.87	10.54 ± 0.75	11.42 ± 0.93	10.62 ± 1.07
		A’ object	13.58 ± 1.35	9.35 ± 1.05	16.18 ± 2.18	11.62 ± 0.73	11.63 ± 0.78	12.21 ± 0.51
		A + A’	27.05 ± 3.05	18.75 ± 2.03^#^	31.76 ± 3.92	22.17 ± 1.24	23.05 ± 1.41	22.83 ± 1.33
NOR TEST	Time spent (s)	A	11.79 ± 1.18	8.58 ± 1.06	9.76 ± 1.57	8.17 ± 0.71	10.67 ± 0.76	10.71 ± 0.96
		B or Novel	19.05 ± 1.91*	12.74 ± 1.56*	15.35 ± 1.81*	10.96 ± 0.81	13.67 ± 0.87	13.62 ± 1.04
		A + B	30.84 ± 2.44^#^	21.31 ± 2.26	25.12 ± 2.99	19.12 ± 1.23	24.33 ± 1.32	24.33 ± 1.81
	Di		0.22 ± 0.05*	0.22 ± 0.07*	0.26 ± 0.07*	0.12 ± 0.07	0.12 ± 0.04	0.13 ± 0.03
MSCF	RAMP	Time spent	69.04 ± 9.63	65.18 ± 11.24	75.90 ± 17.21	82.48 ± 11.52	87.67 ± 11.81	103.53 ± 11.19
		Entries (f)	5.00 ± 0.54	4.50 ± 0.96	5.27 ± 1.07	8.21 ± 1.93	10.21 ± 1.53	11.04 ± 1.43
	BRIDGE	Time spent	12.79 ± 5.37	21.22 ± 9.06	13.48 ± 6.66	40.22 ± 11.07*	68.87 ± 16.81*	54.31 ± 11.06*
		Entries (f)	0.74 ± 0.28	1.20 ± 0.41	0.80 ± 0.24	2.21 ± 0.45	3.54 ± 0.72	3.29 ± 0.61
	CHA	Time spent	16.24 ± 8.44	47.15 ± 14.73	15.97 ± 10.29	90.30 ± 16.36	64.49 ± 16.52	83.53 ± 13.07
		Entries (f)	0.74 ± 0.31	1.30 ± 0.36	0.73 ± 0.36	3.75 ± 0.54	2.58 ± 0.58	3.87 ± 0.57
	SHELTER	Time spent	267.47 ± 35.92	148.37 ± 26.55&	329.80 ± 50.23	155.89 ± 17.81	165.95 ± 18.24	163.26 ± 19.60
		Entries (f)	9.63 ± 0.56	6.95 ± 1.03	10.20 ± 0.74	8.87 ± 0.75	11.00 ± 0.98	9.54 ± 0.87
	OF	Time spent	389.38 ± 24.95	405.12 ± 19.24	335.89 ± 39.11	353.28 ± 18.73	354.99 ± 20.24	376.08 ± 20.15
		Entries (f)	32.79 ± 1.40	29.85 ± 2.17	31.13 ± 3.9	34.21 ± 1.75	37.46 ± 2.09	36.96 ± 2.40
	PASS	Time spent	433.89 ± 17.14	425.02 ± 22.52	416.43 ± 21.36	423.77 ± 18.34	413.34 ± 20.39	374.92 ± 14.13
		Entries (f)	47.84 ± 1.89	42.30 ± 3.62	46.13 ± 4.00	52.42 ± 3.19	58.42 ± 3.24	58.96 ± 3.61

Data gathered in the light-dark box (LDB) test, the novel object recognition (NOR) test and the multivariate squared concentric field (MSCF) test. Data are expressed as mean ± SEM, after collapsing by sex. The latter did not exert, for the most part, significant main effects nor was involved in significant interactions. Thus, to facilitate data presentation, data is collapsed by sex. Di, discrimination index, CHA, challenge area, OF, open-field, PASS, passages. * indicates a significant main effect of AGE (i.e., adolescent vs. adult), in a given variable. # indicates that total time spent in the vicinity of the objects was significantly greater in adolescent rats exposed to i.p. ethanol or to binge ethanol than in the other groups. & indicates that time spent in the SHELTER was significantly greater in adolescents exposed to i.p. or binge ethanol than in controls. Time spent in each section of the MSCF apparatus is expressed in seconds. Please refer to the text for a full account of the significant main effects and significant interactions found. All *p* ≤ 0.05.

#### Light-Dark Box Test

Latency to enter into the black section of the LDB and time spent in the white section (Table 2, upper section) were significantly greater in adolescent than in adults (*F*_1,118_ = 12.72, *p* < 0.001; η^2^*p* = 0.10 and *F*_1,118_ = 32.69, *p* < 0.001; η^2^*p* = 0.22, respectively) yet not affected by the remaining factors, nor the ANOVA yielded significant interactions. Number of transfers (Table 2) was significantly lower in adolescents vs. adults, *F*_1,118_ = 35.75, *p* < 0.001; η^2^*p* = 0.23.

#### NOR Test

**FIGURE 4:**
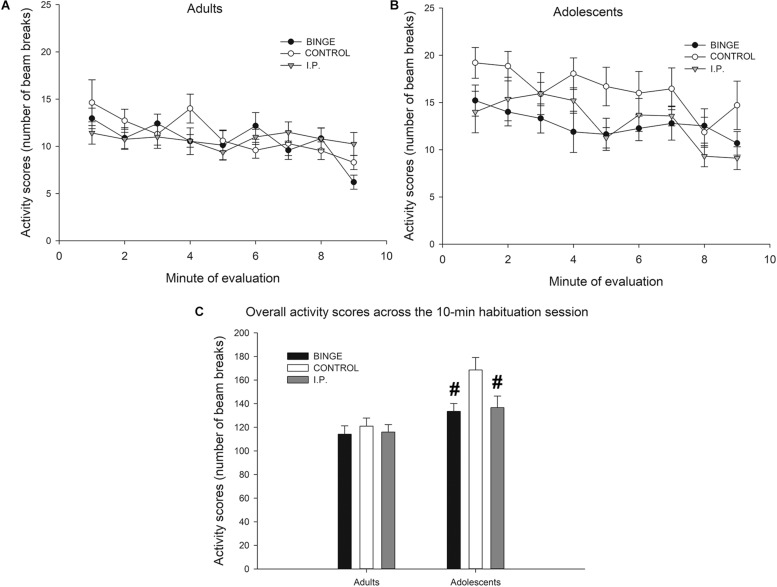
**(A,B)** Activity scores (number of beam breaks) of Wistar rats in an open-field like test conducted at postnatal day 112, as a function of age of first ethanol exposure (adolescence, adulthood; panels **A** and **B**, respectively) and mode of ethanol exposure (BINGE, CONTROL or I.P.). The BINGE treatment consisted of 10 sessions in which the rats were exposed to a bottle of 8% (first two sessions) or 10% ethanol (third and subsequent session) between 1900 and 2100 h. After each session the rats in the IP group were given an intraperitoneal administration of ethanol whose dose was matched for the level of ethanol ingestion exhibited by their same-sex counterparts of the binge group. CONTROL rats were left undisturbed. Sex did not exert a significant main effect upon activity scores, nor was involved in significant interactions. Thus, to facilitate data visualization, data has been collapsed by sex. **(C)** Same data as in upper panel but expressed as total activity counts during the 10-min session. The statistical analysis indicated that the rats first exposed to ethanol as adolescents (either via self-administration or via i.p. administrations) exhibited significantly lower activity scores than same age controls. This effect is indicated in panel **C** via hashtag (#) signs. The BINGE group employed 19 adolescent (9 males, 10 females) and 24 adult (12 males, 12 females) rats, the CONTROL group employed 20 adolescent (12 males, 8 females) and 24 adult (12 males, 12 females) rats, and the IP group employed 18 adolescent (8 males, 10 females) and 24 adult (12 males, 12 females) rats. The data are expressed as mean ± SEM.

The analysis of activity scores during the habituation phase of the NOR protocol revealed significant main effects of Age (*F*_1,118_ = 20.04, *p* < 0.001; η^2^*p* = 0.14, lower activity in adult than in adolescents) and Mode of exposure (*F*_1,118_ = 3.98, *p* < 0.05; η^2^*p* = 0.06). As shown in Figure 4, ethanol exposure – either i.p. or binge drinking – exerted suppressive effects upon activity levels, which seemed specific for adolescents. Separate Mode of Exposure × Age ANOVAs conducted in total activity scores across the 10-min session confirmed this impression. The ANOVA for adults did not reveal significant main effects or significant interactions. In contrast, the analysis for adolescents revealed significantly lower motor activity in ethanol-exposed rats – either via binge drinking or i.p. exposure – than in controls (*F*_2,52_ = 4.06, *p* < 0.05; η^2^*p* = 0.13). Sex did not exert a significant main effect nor was involved in significant interactions. Thus, to facilitate data visualization, data has been collapsed by sex in Figure 4.

During the first phase of the NOR protocol, the rats were introduced in the arena that had explored the day before and were exposed for 5 min to two identical objects (i.e., A and A’). The ANOVA on time spent in the vicinity of the objects at this phase did not reveal a significant main effect of “object,” nor this factor interacted with Sex, Mode of Exposure or Age. This indicated that there was no innate preference for A or A’ across the groups. Yet, the ANOVA and the subsequent tests indicated that total time spent in the vicinity of the objects (i.e., time spent close to A + time spent close to A’) was significantly greater in adolescents, but not adult, rats exposed to i.p. ethanol than in controls (significant interaction between Mode of Exposure and Age, *F*_2,111_ = 4.93, *p* < 0.01; η^2^*p* = 0.08). Adolescents that underwent binge drinking also exhibited a trend (*p* = 0.07) toward greater overall object exploration than controls. These results are in Table 2 (middle section).

During the 5 min NOR test the RM ANOVA (i.e., considering time spent in the vicinity of each object as a repeated measure) yielded a significant main effect of Object and a significant interaction between Object and Age (*F*_1,112_ = 66.30, *p* < 0.001; η^2^*p* = 0.37 and *F*_1,112_ = 7.38, *p* < 0.01; η^2^*p* = 0.06, respectively). As shown in Table 1 and confirmed by the *post-hoc* tests, the rats spent significantly more time near the novel object B than near the familiar object A, an effect that was significantly greater in adolescents than in adults yet was not affected by the history of ethanol exposure (i.e., the interactions comprising Mode of exposure and Object were not significant, all *p* > 0.05). It is worth mentioning, however, that total time spent in the vicinity of the objects (i.e., time spent close to A + time spent close to B) was, in adolescents but not in adults, affected by Mode of exposure. Specifically, the interaction between Age and Mode of exposure achieved significance (*F*_2,112_ = 7.49, *p* < 0.001; η^2^*p* = 0.12) and the *post-hoc* tests revealed that adolescents exposed to binge drinking spent significantly more time at test exploring the objects – regardless their novelty or familiarity – than i.p. or control counterparts. Descriptive data (mean ± SEM) of time spent exploring the objects at the test can be found Table 2.

The ANOVA on discrimination (Di) scores revealed a significant main effect of Age (*F*_1,112_ = 6.02, *p* < 0.05; η^2^*p* = 0.05), with adolescents exhibiting greater Di scores than adults. None of the remaining factors nor the interactions between them achieved significance. Discrimination scores are presented in the middle section of Table 2.

#### MSCF Test

Time spent and frequency of entries in the different sections of the apparatus is shown in Table 2, lower section. The ANOVA of overall locomotor activity during the test – i.e., total frequency of transfers between compartments – revealed a significant main effect of age (*F*_1,__114_ = 17. 70, *p* < 0.001, η^2^*p* = 0.13; i.e., greater motor activity in adult than in adolescent rats) that did not interact with the other factors. This indicates that time spent in the different sections of the maze was not affected by ethanol-induced alterations in motor activity.

An important result was that shelter seeking was not affected by mode of ethanol exposure in those rats had been exposed to ethanol as adults, yet it was significantly greater in adolescents exposed to i.p. or binge ethanol than in controls (significant age × treatment interaction: *F*_2,114_ = 4.95, *p* < 0.001, η^2^*p* = 0.08, descriptive data shown in Table 2). More in detail, IP or BINGE adolescents exhibited a circa two-fold increase in time spent in the dark and enclosed SHELTER. Time spent in these sections was also greater in females than in males (*F*_1,114_ = 9.98, *p* < 0.001, η^2^*p* = 0.08).

Time spent in the BRIDGE (*F*_1,114_ = 16.99, *p* < 0.001, η^2^*p* = 0.13) was significantly greater in adults than in adolescents, a pattern also found among female rats in terms of time spent in the RAMP or CHA areas (significant sex × age interaction, *F*_1,114_ = 4.56, *p* < 0.05, η^2^*p* = 0.04 and *F*_1,114_ = 7.06, *p* < 0.001, η^2^*p* = 0.06, respectively). Time spent in the OF was lower in IP females than in IP males, yet similar among male and females given binge or i.p. ethanol exposure (significant sex × treatment interaction, *F*_2,114_ = 5.07, *p* < 0.01, η^2^*p* = 0.08).

### Two-Bottle Choice Ethanol Intake Tests Conducted at Late Adulthood (Exp. 1)

The long-term effects of age of first ethanol exposure and the mode of exposure of such experience (i.e., 10 binge or i.p. ethanol exposures plus two 24 h choice tests between ethanol and water or – CONTROL group – only the two 24 h choice tests) were assessed in 24 h-long, two-bottle, ethanol intake tests. These tests took place at late adulthood, on PDs 120–139.

**FIGURE 5:**
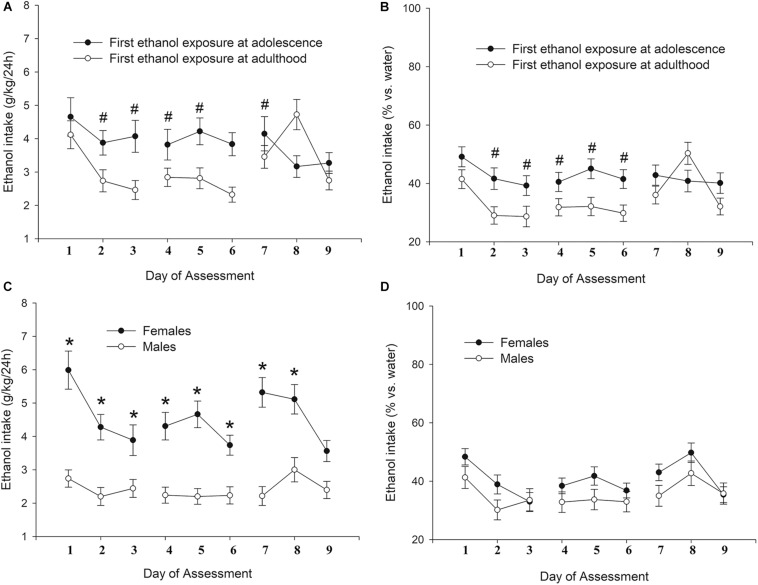
Ethanol intake (g/kg and percent preference, panels **A** and **C**, and panels **B** and **D**; respectively) in Wistar rats as a function of sex (females, males, panels **C**,**D**) or age of first ethanol exposure (adolescence, adulthood; panels **A**,**B**) and day of assessment (9 intermittent two-bottle intake tests conducted at PDs 120–139). Data in panels **A** and **B** is present collapsed across sex, whereas data in panels **C** and **D** is presented collapsed across age of first ethanol exposure. The statistical analyses indicated that the rats that had been first exposed to ethanol at adolescence exhibited, when compared to those that had been first exposed as adults, significantly greater ethanol intake and ethanol percent predilection at sessions 2, 3, 4, 5, and 6. These significant differences are indicated by the hashtag (#) sign. The analyses also indicated that female rats drank more (g/kg) ethanol than male rats in sessions 1–8, which is indicated by the asterisk sign. There were 57 rats first exposed to ethanol at adolescence (29 males, 28 females), whereas 72 were first exposed to ethanol as adults (36 males, 36 females). The data are expressed as mean ± SEM.

The ANOVA for g/kg ingested yielded significant main effect of Age of first exposure (*F*_1,115_ = 6.90, *p* < 0.01; η^2^*p* = 0.57) and significant Sex × Day, and Age × Day interactions (*F*_8,920_ = 3.06, *p* < 0.05; η^2^*p* = 0.03 and *F*_8,920_ = 4.97, *p* < 0.001; η^2^*p* = 0.04, respectively). The ANOVA for percent ethanol predilection, in turn, revealed significant main effects of Age of first exposure and Day (*F*_1,115_ = 4.73, *p* < 0.05; η^2^*p* = 0.04 and *F*_8_,_920_ = 5.94, *p* < 0.001; η^2^*p* = 0.05, respectively) and a significant Age of first exposure × Day interaction (*F*_8,920_ = 3.71, *p* < 0.001; η^2^*p* = 0.03). The *post-hoc* tests indicated that the rats that had been exposed, and thus initiated to ethanol, to the BINGE/IP/CONTROL procedures at adolescence exhibited greater ethanol intake and ethanol percent predilection than those that had been exposed as adults, an effect that achieved significance at sessions 2, 3, 4, 5, and 6. Also, female rats drank more ethanol (g/kg) than male rats in sessions 1–8. Figure 5 depicts absolute and percent ethanol intake as a function of age of first ethanol exposure, and day of assessment. The upper panels (A,B) present the data collapsed across sex, whereas the lower panels (C,D) present the data collapsed across age of first ethanol exposure.

### Binge Drinking After Naloxone Administration (Exp. 2a), Two-Bottle Choice Ethanol Intake Tests Conducted Before and After Binge Drinking Exposure (Exp. 2a) and BELs Registered on Binge Session 3 (Exp. 2b)

**FIGURE 6:**
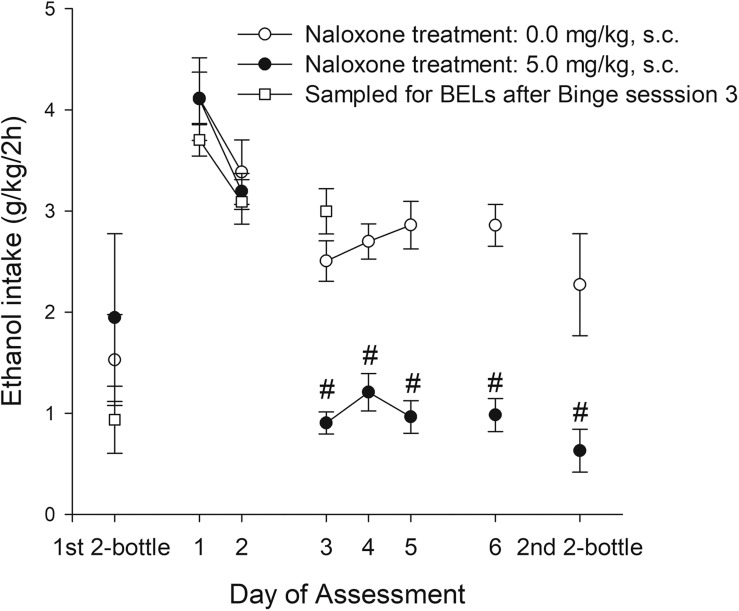
Ethanol intake (g/kg) in male adolescent rats of Experiment 2a and 2b. Data for Experiment 2a is presented as a function of binge intake session (i.e., Day of Assessment 1–6) and naloxone treatment (0.0 or 5.0 mg/kg, subcutaneous, s.c.) applied 30 min before commencement of binge sessions 3–6. On each binge session the rats were exposed to a bottle of 8% (first two sessions) or 10% ethanol (third and subsequent sessions) between 1900 and 2100 h. The rats were assessed on a two-bottle, 24 h free-choice, test on PD 30 (pre-test before exposure to binge) and a post-test conducted 48 h after termination of the binge-like exposure. The statistical analyses indicated that, on binge sessions 3–6 and on the two-bottle choice test conducted after the binge exposure, the rats given 5.0 mg/kg naloxone drank significantly less than dose given vehicle. These significant differences are indicated by the hashtag (#) sign. Twenty rats were employed (10 administered naloxone, 10 administered vehicle). The figure also depicts (i.e., white squares) mean ethanol intake (g/kg) achieved in six male adolescent rats (Exp. 2b) that underwent the first two-bottle choice test and three 2-h binge sessions. These rats were decapitated at termination of binge session 3 and blood samples were obtained and processed for blood ethanol levels. The data are expressed as mean ± SEM.

Experiment 2a exposed adolescent rats to six sessions of the binge protocol. In sessions 1 and 2 they were given vehicle administration prior to the test, whereas in sessions 3–6 they were administered naloxone, 30-min prior to the 2-h access to ethanol. Two-bottle ethanol intake tests (length: 24 h) were conducted 48 h before and 48 h after the binge protocol. The ANOVA for g/kg ingested during the binge protocol revealed significant main effects of naloxone and a significant interaction between Naloxone and Session (*F*_1,18_ = 37.03, *p* < 0.001; η^2^*p* = 0.67 and *F*_5,90_ = 8.86, *p* < 0.001; η^2^*p* = 0.33, respectively). As shown in Figure 6 and confirmed by the pair-wise comparisons, ethanol intake was similar across all rats in sessions 1 and 2 (i.e., when all rats were treated with vehicle), yet on sessions 3–6 the rats given naloxone drank significantly less than dose given vehicle. Naloxone administration was associated with a 2-fold reduction in ethanol intake. Ethanol ingestion during the baseline two-bottle choice test was similar in the rats that would be treated with naloxone or vehicle. In contrast, ethanol intake at the two-bottle choice test conducted after the binge exposure revealed significantly lower ethanol intake in rats treated with naloxone during the binge vs. those given vehicle (*t*_16_ = 2.75, *p* < 0.05). There was no drug administration immediately before these tests.

Experiment 2b exposed six male adolescent rats to a two-bottle choice between 8% ethanol, followed by two 2-h binge sessions in which they had access to 8% ethanol. At binge session 3 they drank 10% ethanol before being decapitated. Mean ethanol intake (g/kg) achieved during each measurement is depicted in Figure 6. The mean BEL registered at the end of binge session 3 was 60.82 ± 22.39 mg/dl. There was a positive and significant correlation between the BELs registered at the end of binge session 3 and the g/kg ingested by the rats during that session (*r* = 0.87, *p* < 0.05).

## Discussion

A main result was the dramatic difference in binge-like ethanol drinking between adolescent and adult rats. Up to a 3-fold difference was observed between these groups, an effect most noticeable in males than in females and in the initial than in the latter binge sessions. The results agree with epidemiological studies indicating that adolescents drink less often than adults, yet when they do they ingest significantly greater quantities (Windle and Zucker, 2010). Specifically, it has been shown that adolescents drink more than twice as much than adults per drinking occasion (Substance Abuse and Mental Health Services Administration, 2006). A nationally representative study reported, the ingestion of 38.8 and 80.1 g of alcohol per consumption episode in individuals aged ≥65 or 14–24 years respectively (Servicio Nacional para la Prevención y Rehabilitación del Consumo de Drogas y Alcohol, 2017).

Pre-clinical studies also suggest that, under different conditions and settings, adolescents drink more than adults, albeit the evidence is much less abundant in rats than in mice (Doremus et al., 2005). For instance, C57BL/6J adolescent mice given DID-like ethanol access (2 h per night) consumed significantly more than their adult counterparts, an effect that persisted 3 weeks later, when both groups of mice were adults (Moore et al., 2010). Yet, the results are far from being conclusive. In the latter study DBA/2J mice showed no adolescent vs. adult difference, neither in the initial DID phase nor in the second phase conducted 3 weeks later. Another study (Younis et al., 2019) applied the DID procedure in adolescent or adult C57BL/6J mice and found, unlike our work, similar drinking of 20% ethanol across 9 (i.e., between 6 and 8 g/kg/4 h).

DID-like or scheduled access to ethanol has been much less employed in rats, albeit some success has been achieved when using lines selectively bred to show innate preference for ethanol (Bell et al., 2014). Nowak et al. (1999) and McKinzie et al. (1998) reported that adult female or male, alcohol-preferring (P), rats consumed ∼2 g/kg in 2 h-long sessions conducted during the dark phase. Sardinian alcohol-preferring rats, on the other hand, drank ≤1.0 g/kg ethanol when the 2 h drinking sessions occurred immediately after lights off (Colombo et al., 2017). These levels of ethanol consumption, achieved by adult rats derived from lines selected for high alcohol consumption, are generally lower than those found in the genetically heterogeneous adolescents of the present study, which drank 2.5–3.0 g/kg/2 h in the first week of limited, binge-like, access to ethanol and 2.0–2.5 g/kg/2 h in the subsequent weeks. Interestingly, the latter levels are similar to those reported by Bell et al. (2011) in adolescent P rats exposed to a limited access binge-like procedure.

Also interesting is that the greater DID-like drinking reported in adolescent vs. adult rats or mice seems particularly noticeable in social situations. Logue et al. (2014) reported very little adolescent vs. adult mice differences in a short (45 min, housed one animal per cage) session of access to 5% ethanol, yet the adolescents drank significantly more if tested with a companion. Our study, conducted in rats, did not systematically vary social conditions, yet the animals were tested in their homecage and separated from the partner via a lid that prevented touching but not smelling or hearing. It is possible that these conditions favored greater ethanol intake in the youth vs. the adults.

The greater binge drinking of the adolescents was particularly noticeable during the early sessions, yet as testing progressed they merged their level of intake with that shown by adults. More in detail, binge-like consumption decreased over time in the adolescents, an effect particularly noticeable in male rats. These results seem to clash with those from studies [for review and references, see Carnicella et al. (2014)] suggesting an escalation of ethanol consumption in rats when employing intermittent alcohol exposure protocols, commonly referred to as intermittent access to ethanol in 2-bottle choice [IA2BC, e.g., Maier et al. (2019)]. The latter literature, however, has focused on adult rats, whereas the result we are discussing was exhibited by adolescent subjects. Interestingly, the patterns displayed by these adolescents are reminiscent of those reported by Bell et al. (2011) and by Truxell et al. (2007). The latter authors exposed adolescent or adult rats to the consumption-off-the floor paradigm, in 3-daily sessions spread across a 5-day period, and observed that ethanol ingestion significantly decreased over time in so-called juveniles (i.e., tested at PDs 25–28) or in adolescents tested at PDs 30–34, yet ethanol ingestion remained stable in young adult rats tested at PDs 60–64. It is thus possible that the pattern displayed by the adolescent rats, in the DID-like section of the present study, may reflect a normative decrease in ethanol acceptance, as the animals transition from adolescence to adulthood. It is worth noting that, during the course of the protocol, we employed increasing concentrations of alcohol (from 8 to 10%), yet these were still lower than those employed in other DID-like procedures [e.g., 20% (Pavon et al., 2016)]. Perhaps different ethanol self-administrations patterns would have been observed had we employed higher ethanol concentrations, or had we kept the ethanol concentration stable across sessions.

Another aim was to analyze effects of the binge-like ethanol exposure upon exploratory and anxiety responses, and cognitive performance. It has been shown that ethanol administration (3.0–5.0 g/kg, i.p.) throughout adolescence impairs conditioned discrimination learning (Pascual et al., 2007), reversal spatial learning in the Morris water maze (Coleman et al., 2011), and short-term recognition memory in the NOR test and in an odor-habituation test (Montesinos et al., 2015). Some studies suggest that rats or mice given similar treatments at adulthood are spared from these effects. A study (White et al., 2000) gave rats 5.0 g/kg i.p. ethanol every other day over 20 days, beginning at PD 30 or 70. At a subsequent test in a radial arm maze, the rats treated with ethanol at adolescence – but not those treated at adulthood – exhibited working memory impairments. Short- and long-term spatial memory, however, was unaffected, as well as anxiety responses in an EPM. Similar lack of alterations in anxiety response after adolescent or adult i.p. binge exposure were observed in Wistar rats (Fabio et al., 2014).

In the present study the effects of binge ethanol exposure upon cognitive or exploratory responses were observed only when ethanol exposure occurred at adolescence. Ethanol exposure at adolescence, either i.p. or binge, significantly reduced the exploration of the open field-like chamber in which the NOR training took place. Reduced propensity to explore novel environments suggests an anxiety-like profile, and has been found after stress (Berridge and Dunn, 1986; Pautassi et al., 2012). The possibility that ethanol exposure induced an anxiety-prone phenotype in adolescents, but not in adults, is consistent with the finding that shelter-seeking in the MSCF test was significantly increased in adolescents exposed to i.p. or binge ethanol, but not altered in control adolescents or in adults.

Repeated ethanol administration (2.0–4.0 g/kg, once daily for a total of 7–8 administrations) has been shown to impair cognitive performance in the NOR test in rats (Marszalek-Grabska et al., 2018) and in mice (Wolstenholme et al., 2017). Moreover, Marco et al. (2017) reported performance deficits in the NOR test, in male and female Wistar rats that self-administered ethanol (20% in drinking water) four times a week during PDs 28–52. Unlike these studies, in the present work novelty object recognition was preserved after ethanol exposure, with adolescents and adults preferring the novel over the known object. Discrimination scores, however, were significantly higher in adolescents than in adults, a result probably obeying to the greater levels of novelty preference normatively exhibited by adolescent, when compared to adults (Stansfield and Kirstein, 2006; Walker et al., 2017). Binge- or experimenter-administered, ethanol-induced, alterations could probably have been observed if we had employed the spatial variant of the NOR test. The latter, but not the NOR, test is sensitive to hippocampal alterations (Jablonski et al., 2013), and we (Fernandez et al., 2019) and others (Hunt and Barnet, 2016) have shown that ethanol treatments akin to those of the present study yield alterations in the adolescent – but not in the adult – hippocampus of the rat.

Our ethanol-exposed adolescents, however, did show alterations during the NOR protocol, in terms of the overall level of exploratory activity. Specifically, during the familiarization phase time spent in the vicinity of the objects was greater in IP or BINGE adolescents than in CONTROLS. This effect was also observed during the NOR test, albeit in the BINGE group only. This result indicates that adolescent ethanol exposure affected exploratory patterns in the NOR test, although this effect did not translate to alterations in cognitive performance (i.e., novelty object recognition was preserved after ethanol exposure).

As expected, the binge exhibited by the adolescents was blocked by acute pre-treatment with naloxone. The blockade of the opioid system reduces, in humans, the enhancement in mood ratings found the after drinking ethanol (Davidson et al., 1999) and, in rats, blocks ethanol-induced behavioral stimulation, ethanol-induced conditioned place preference (Pautassi et al., 2011) and ethanol drinking (Shoemaker et al., 2002). An interesting result of Experiment 2a was that naloxone had a lingering effect, reducing ethanol drinking vs. vehicle-treated controls at the 2nd two-bottle choice test, long after its clearance. An important limitation was that we did not assess naloxone effects upon baseline water ingestion, which detracts from the specificity of the effect reported in Experiment 2a. Moreover, the binge pattern reported was observed after a mild (50% of the water usually consumed by the rats) yet significant water restriction. We did not include a group of rats that had access to the binge sessions without water restriction, so we can not dissect the influence of this procedural factor.

In Exp. 1, greater binge drinking at adolescence did not enhance 24 h 2-bottle choice drinking, neither when tested immediately after the binge sessions nor at adulthood. Age of first exposure to ethanol, however, did affect level of intake during the tests conducted at late adulthood, with rats that had been initially exposed to ethanol at adolescents drinking significantly more than those that had similar exposure during early adulthood. This permissive effect of adolescent ethanol exposure upon adult ethanol drinking was similar in IP or BINGE groups, indicating that the effect was not dependent on the intensity of such exposure; and emerged even after the brief experience with the drug of CONTROL subjects, which were exposed to ethanol during the two 24-h choice tests. In other words, adolescent binge ethanol exposure did not enhance later, free-choice, drinking at adulthood to a greater length than i.p exposure. Instead, it seems that any kind of adolescent exposure to ethanol during adolescence, even the brief level experienced by those in the CONTROL condition, is sufficient to enhance the proclivity to ingest and prefer the drug, when compared to subjects given similar ethanol exposure but during adulthood. These results agree with clinical and pre-clinical work that argue in favor of the “early debut” effect (Vera et al., 2019; Younis et al., 2019), yet differ from studies that suggest that this effect is more likely to be expressed by those that experienced levels of intoxication consistent with a drunkenness or binge episode (Kuntsche et al., 2013; Fabio et al., 2014).

It could be argued that none of the adolescents in our study achieved levels of intoxication compatible with the definition of binge, as the mean BEL registered at the termination of binge session 3 in Exp. 2b (i.e., 60.82 ± 22.39 mg/dl) was shy from the 80 mg/dl threshold commonly used to define binge drinking (e.g., Hosova and Spear, 2017). Yet it should be noted that we measured BELs 120 min after the commencement of the binge, and others have suggested that schedules of restricted drinking induce most of the intake during the initial 30–60 min (McKinzie et al., 1998; Nowak et al., 1999). BELs also had the confound of being assessed after ethanol exposure (i.e., the two-bottle choice tests the binge sessions 1 and 2).

Another relevant methodological detail is that we equated the age of testing for both group of rats (i.e., those exposed to alcohol as adolescents or as adults) and tested them at the same age at adulthood. A caveat of this procedure is that the delay between the last two-bottle choice session at the first exposure phase and the first two-bottle test at adulthood is different between the two age groups (i.e., substantially shorter for those exposed to alcohol for the first time as adults). This confound was also present during the behavioral assessments at PDs 110–113 (i.e., LDB, MSCF, and NOR tests). It is possible that lingering withdrawal effects were still present at that moment and, thus, affected anxiety-like responses differentially in adolescent and adults.

A dissociation was observed in regards with the effect of sex on ethanol intake. During the two-bottle intake tests ethanol intake was significantly greater in females than in males, regardless age; yet ethanol intake during the binge drinking sessions was affected by sex (i.e., significantly greater in females than in males) in adult rats only. It is possible this dissociation obeys to female adolescent rats exhibiting a functional ceiling effect during the binge sessions, that prevented them from exhibiting their (relative to males) high-drinking phenotype (Li et al., 2019). It should be noted that the greater consumption of the females on the 1st evaluation of ethanol intake may have had carry-over effects, affecting the drinking levels observed afterward. Greater ethanol intake in female than in male rats is a consistent finding in pre-clinical research, yet the emergence of the phenomenon is affected by testing conditions. For instance, a study that employed the two-bottle choice test reported (Penasco et al., 2015) greater ethanol intake in female than in male Wistar rats, yet only after exposure to a week of alcohol cessation combined with restraint stress.

Another limitation of the study is that, in Experiment 1, the significantly greater ethanol consumption observed in adolescents vs. young adults occurred in rats that were water deprived and only had access a to single ethanol bottle. It certainly conceivable that these results obey, at least partially, to age-related differences in thirst (Kenney and Chiu, 2001). This possibility cannot be discarded. It is important to remark, however, that baseline water consumption during the two-bottle choice intake test conducted before binge drinking exposure indicated that water intake per gram of body weight was higher in adult than in adolescent rats. This effect of greater water intake in adult than in adolescent rats was also observed during the *post-test* intake session, which took place after binge exposure.

Effect sizes of the most relevant significant main effects or significant interactions reported were relatively variable. Most of the significant effects reported for the analysis of ethanol intake scores were medium size (i.e., η^2^*p* = 0.06–0.13), albeit the reduced *post-test* ethanol intake observed in BINGE and IP groups (relative to controls) was associated with a big effect size. A big effect size was also found for naloxone’s consequences upon ethanol intake (η^2^*p* = 0.33). Effect sizes for the significant main effects or significant interactions reported for the variables measured at the LDB, MSCF, or NOR tests were also highly variable, with most falling within the medium size effect range.

Despite the limitations, the study cements the notion that binge-like ethanol drinking is substantially greater in adolescent that in adults, an effect that can be normalized by blockade of opioid transmission. Ethanol exposure at adolescence, but not at adulthood, was associated with altered response to novel stimuli and greater subsequent ethanol intake at late adulthood. The results support the notion that preventing alcohol access to adolescents should reduce the likelihood of problematic alcohol use and alcohol-related consequences.

## Data Availability Statement

The datasets generated for this study are available on request to the corresponding author.

## Ethics Statement

The animal study was reviewed and approved by Institutional Animal Care and Use Committee at INIMEC-CONICET-UNC (CICUAL).

## Author Contributions

ASa, ASu, LR-L, and RP run the intake tests at adolescence and adulthood. RP, CC, IM, ASu, ASa, and LR-L had the original scientific idea, designed the study and analyzed the data. ML run the behavioral assays and analyzed that section of the data. LR-L run Experiment 2b and processed the blood samples. ASu, RP, and LR-L wrote the initial draft of the manuscript. All authors participated in the subsequent writing of the manuscript and gave approval to the final form.

## Conflict of Interest

The authors declare that the research was conducted in the absence of any commercial or financial relationships that could be construed as a potential conflict of interest.
